# Enhancing interpretability and accuracy of AI models in healthcare: a comprehensive review on challenges and future directions

**DOI:** 10.3389/frobt.2024.1444763

**Published:** 2024-11-28

**Authors:** Mohammad Ennab, Hamid Mcheick

**Affiliations:** Department of Computer Sciences and Mathematics, University of Québec at Chicoutimi, Chicoutimi, QC, Canada

**Keywords:** artificial intelligence, machine learning, deep learning, healthcare, interpretability, explainability, accuracy

## Abstract

Artificial Intelligence (AI) has demonstrated exceptional performance in automating critical healthcare tasks, such as diagnostic imaging analysis and predictive modeling, often surpassing human capabilities. The integration of AI in healthcare promises substantial improvements in patient outcomes, including faster diagnosis and personalized treatment plans. However, AI models frequently lack interpretability, leading to significant challenges concerning their performance and generalizability across diverse patient populations. These opaque AI technologies raise serious patient safety concerns, as non-interpretable models can result in improper treatment decisions due to misinterpretations by healthcare providers. Our systematic review explores various AI applications in healthcare, focusing on the critical assessment of model interpretability and accuracy. We identify and elucidate the most significant limitations of current AI systems, such as the black-box nature of deep learning models and the variability in performance across different clinical settings. By addressing these challenges, our objective is to provide healthcare providers with well-informed strategies to develop innovative and safe AI solutions. This review aims to ensure that future AI implementations in healthcare not only enhance performance but also maintain transparency and patient safety.

## 1 Introduction

In recent years, AI technology has enabled the widespread adoption of machine learning (ML) and deep learning (DL) methods in healthcare. These techniques have automated various processes like screening, diagnostics, and treatment (Zhongqi and Jia, 2022). However, DL algorithms are often considered “black boxes” due to their intricate training and numerous parameters, making it hard to decipher the connection between inputs and outputs (Zhang and Zhang, 2023). Although advanced DL methods can significantly improve speed and accuracy without human intervention, establishing an interpretable AI framework remains imperative. Understanding algorithms’ inner workings and prediction bases is essential, not just competitively but also for stakeholder trust (Stinson and Vlaad, 2024). Discussing trustworthy AI involves the terms accuracy, interpretability, and explainability, which are occasionally used interchangeably. In data mining and machine learning, interpretability is the interface between people and decision models that accurately represents the model and is intelligible to humans (Savindu Herath Pathirannehelage et al., 2024).

## 2 Research gaps

While current literature highlights the use of AI in healthcare, the research gaps in terms of model generalizability, lack of real-time interpretability, and inclusion of diverse clinical data remain relatively unexplored. Additionally, the integration of uncertainty quantification with interpretability models is an underexamined area that could improve AI adoption in healthcare settings. Gaps related to real-time feedback for clinicians and the role of user-centered design in AI development also need further exploration (Helman et al., 2022).

This paper characterizes the challenges of AI applications in healthcare focused on interpretability and accuracy to ensure accountability and regulatory compliance. Our survey helps healthcare providers develop appropriate strategies to rapidly implement innovative solutions safely. The goal is consolidating existing knowledge on AI systems and the interpretability models to help researchers swiftly grasp the state-of-the-art and determine areas needing more research (Saeed and Omlin, 2023). We also distinguish our review from earlier studies by focusing on both the technical and practical limitations of AI in healthcare, which are often overlooked in previous reviews, such as those focusing solely on specific AI techniques or healthcare applications.

The primary contributions of this work include:

• Summarizing the current state of AI systems in healthcare.
• Identifying and detailing the key limitations encountered when developing AI systems, with a focus on both interpretability and accuracy.
• Discussing the research gaps and future challenges in AI application in healthcare.
• Offering a detailed comparison of how this review differs from previous literature, including works like (Sadeghi et al., 2024).

Additionally, we consider the control strategy presented by Stefanelli et al. (2023) in their work on sensorimotor control in prosthetics, which integrates both force and temperature information to replicate human reflexive behavior during manipulation. This approach provides valuable insights into designing more human-like AI systems in healthcare by considering multimodal sensory inputs.

## 3 Methodology

To achieve our objectives, we systematically reviewed 61 AI systems for classifying and treating various diseases in healthcare. The selected systems were analyzed by extracting how they address the challenges of assessing AI systems in terms of interpretability and accuracy. Our work defines the black box of healthcare AI applications, emphasizing model interpretability and accuracy.

### 3.1 Search strategy

We conducted our search using multiple databases, including Google Scholar, PubMed, and IEEE Xplore, to ensure comprehensive coverage of the literature. The search was conducted using the following keywords, combined with Boolean operators:

• “Artificial Intelligence” AND “Healthcare” AND “Accuracy”
• “Deep Learning” AND “Interpretability” AND “Healthcare”
• “Machine Learning” AND “Explainability” AND “Healthcare”

### 3.2 Inclusion criteria

• Peer-reviewed articles focused on healthcare AI applications.
• Studies published between 2010 and 2024.
• Articles emphasizing interpretability and/or accuracy (both ML and DL).

### 3.3 Exclusion criteria

• Articles not focused on AI techniques (i.e., non technical).
• Non-healthcare AI applications.
• Legislative or legal discussions that do not contribute to the technical understanding of interpretability.

### 3.4 Data extraction

For each selected study, we extracted information on:

• The AI model used.
• The metrics employed to assess interpretability and accuracy.
• The context in which the AI model was applied.
• A detailed discussion on the results achieved.

The results of the literature analysis are summarized in Table 1.

**TABLE 1:** Summary of selected models that applied AI models in various healthcare contexts.

Study	AI model	Accuracy metric	Interpretability	Context	Results
Zhongqi and Jia (2022)	Deep Learning	95%	Black-box	Diagnostic Imaging	High accuracy but limited interpretability
Jing et al. (2023)	Neural Networks	92%	Explainable AI (XAI)	Predictive Modeling	Improved performance with *post hoc* explanation methods
Stinson and Vlaad (2024)	Deep Neural Networks	97%	None	Screening and Diagnostics	Excellent accuracy but no real-time interpretability
Helman et al. (2022)	Random Forests	89%	Global Interpretability	Treatment Decision Support	High interpretability but moderately lower accuracy
Mella et al. (2023)	Support Vector Machines	91%	Local Interpretability	Diagnostics	High accuracy with interpretable decision boundaries
Hamm et al. (2023)	Deep Learning	95%	Available	Prostate cancer	High accuracy
Subramani et al. (2023)	Deep Learning	96%	Black-box	Cardiovascular diseases	High accuracy
Nicolae et al. (2020)	Machine Learning	Treatment Dose Planning	Black-box	Rectal cancer	Effective planning
Nicolae et al. (2020)	Machine Learning	Effectively reduce planning time	Black-box	Prostate cancer	Efficient planning
Afrash et al. (2023)	Deep Learning	83%	Black-box	Gastric cancer	High accuracy
Subramani et al. (2023)	Deep Learning	96%	Black-box	Cardiovascular diseases	High accuracy

**FIGURE 1:**
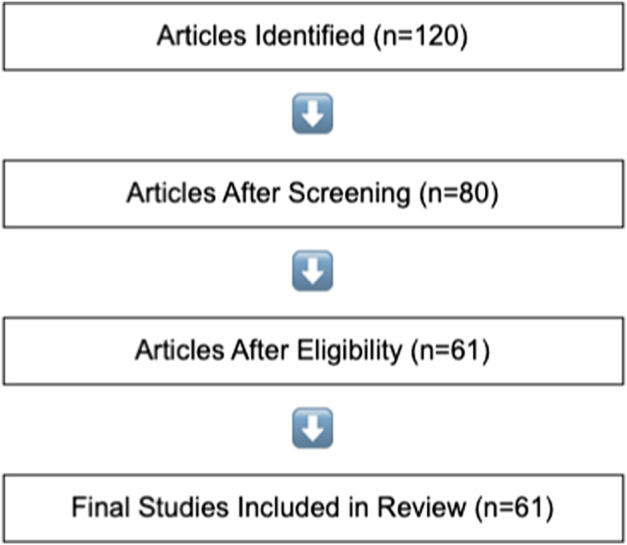
PRISMA diagram illustrates the article selection process.

The PRISMA diagram shown in Figure 1 illustrates the article selection process:

### 3.5 Study limitations

Our review primarily focuses on AI models applied in diagnostic imaging tasks. While we have considered various healthcare applications (e.g., screening, and treatment decision-making), our scope does not extend to other domains like genomics or personalized medicine, which could benefit from AI innovations. Additionally, the lack of real-time applicability and the high computational cost of certain models have not been deeply explored in this review.

## 4 Summary of selected literature

We reviewed 61 papers that applied AI models in various healthcare contexts. Table 1 summarizes the key information extracted from each paper, including the AI model used, the metrics for accuracy and interpretability, and the results achieved.

## 5 Key metrics in AI model development

Despite the numerous benefits, AI models in healthcare are not without limitations. These include:

### 5.1 The accuracy

Accuracy is paramount in healthcare applications, especially for tasks like diagnostic imaging and predictive modeling. AI systems can often surpass human experts in detecting abnormalities (Esteva et al., 2017). However, the performance of AI models is typically evaluated using historical data, split into training and testing sets. This method, though widely used, does not always reflect real-world performance (Baeza-Yates, 2022). The trade-off between model complexity and accuracy is an ongoing challenge.

### 5.2 The interpretability

Interpretability in AI refers to the degree to which a human can understand and trust the decisions made by AI systems. In the context of healthcare, interpretability is paramount because medical professionals rely on these systems to make critical decisions about patient care. Without a clear understanding of how AI systems arrive at their conclusions, healthcare providers may be reluctant to adopt these technologies or may misinterpret their recommendations, potentially leading to adverse patient outcomes (Ennab and Mcheick, 2022b). Techniques like activation visualization enable us to infer which features in an image are represented by each feature map within every layer of the trained convolutional neural network. When activation visualization is needed, it involves inputting sample medical images into the convolutional neural network for analysis and observing the patterns in the results (Kim, 2023).

### 5.3 The interpretability-accuracy trade-off

As highlighted (Ennab and Mcheick, 2022a), there is often a trade-off between interpretability and accuracy. While simpler models like decision trees are more interpretable, they may not achieve the same level of accuracy as more complex models, such as deep neural networks (Escalante et al., 2018).

## 6 AI models in healthcare

**FIGURE 2:**
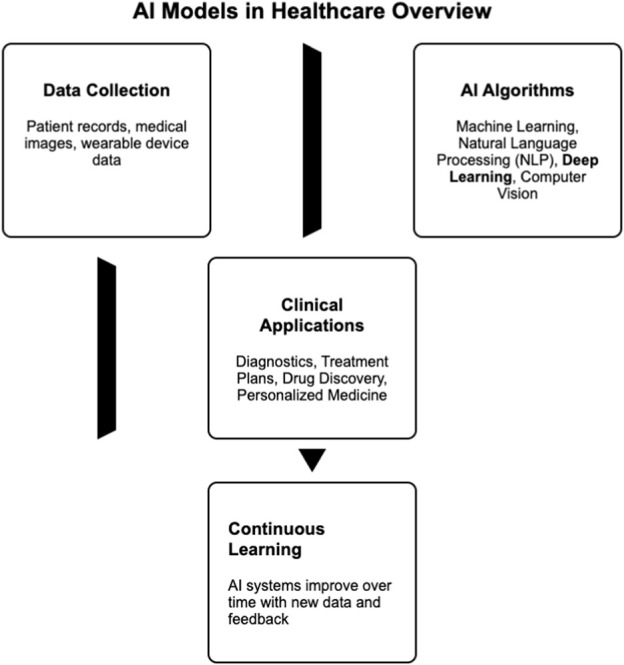
A diagram illustrating AI models in healthcare.

The field of medicine has employed AI technology to automate various stages of clinical research, offering valuable assistance for clinical decision-making (Wu Yazhou and Chen, 2022). Utilizing AI techniques across diverse medical domains brings advantages such as enhancing diagnostic accuracy and reducing both time and labor requirements. Leveraging the latest AI advancements, typical applications experiencing revolutionary changes include intelligent screening, precise diagnosis, risk prediction, and supportive therapy (Khalifa et al., 2024) as shown in Figure 2.

### 6.1 Conversational AI

Conversational AI is a technology that leverages conversation-oriented techniques, facilitating dynamic interactions and widespread engagements across various users and platforms (Kaushik, 2023). It refers to a set of technologies that communicate with humans using “natural language” through hardware, software, etc., by combining natural language processing (NLP), ML, DL, and conversation processing technologies into a single platform (Han et al., 2023). Within the healthcare sector, conversational AI is facilitating several significant use cases that have an impact on both healthcare providers and patients. In healthcare, five distinct use cases have been recognized:

(1) Disseminating Information: AI assistants can engage in conversational interactions to provide answers to frequently asked questions (FAQs) concerning specific illnesses, health conditions, or pandemics (Haptik, 2023). It has the potential to increase awareness of a specific health-related issue or disaster by providing quick access to accurate, trustworthy, and timely information, e.g., Dr. LalPathLabs (Gottlieb et al., 2023).
(2) Scheduling appointments: Conversational AI enables patients to easily arrange and reschedule medical appointments (Haptik, 2023).
(3) Patients care: Healthcare organizations have effectively used AI Assistants to automate the answer to frequently asked questions and the resolution of regular, repetitive chores and diagnostics chains (Haptik, 2023).
(4) Managing and tracking patients’ health: conversational AI aids individuals in monitoring their physical wellbeing by detecting symptoms at an early stage and facilitating online consultations with healthcare professionals when necessary (Haptik, 2023). Additionally, patients are equipped with the resources and information required to manage their own health effectively (Han et al., 2023).
(5) Enhancing Patient Assistance: conversational AI fosters a data-driven approach within healthcare, empowering patients and caregivers with the necessary information for informed decision-making (Haptik, 2023).

### 6.2 Intelligent screening

AI technology has been applied in the screening of various malignant tumors, and it can automatically screen the beginning and malignant areas of suspected cancer. However, it should be noted that the accuracy of the model has a major impact on the clinical decision-making of physicians (Chen et al., 2024). When the model’s predictions are inaccurate, the effectiveness of its supplementary screening is frequently significantly diminished. Moreover, for diseases with low occurrence rates and limited sample sizes, the presence of false positives cannot be disregarded, making it advisable to conduct manual reviews for verification. Consequently, considerable challenges persist in the application of AI models in clinical settings, and it is essential to factor in the potential adverse repercussions of model-assisted screening during the design of AI tools. Furthermore, it is noteworthy that all AI systems operated as black boxes without real-time interpretability techniques (Wu Yazhou and Chen, 2022).

### 6.3 Screening for digestive tumors

Kiani et al., (2020) developed a deep learning system for liver pathology image analysis; hence, the system can be self-employed in the screening of both hepatocellular carcinoma and cholangiocarcinoma. When tested against the validation set, this system has a performance accuracy of 88.5% and boasts the same accuracy of 88.5% for the independent test set. A similar system was developed by Sinha et al., (2024), which carried out the analysis of colonoscopy images with the help of machine learning. Precisely, the system detects and differentiates the adenomas, which need to be extracted, from nonneoplastic polyps, while having a predictive accuracy of 98.1%. A similar artificial intelligence (AI)-based deep learning system was developed by Wang et al., (2020) for colonoscopy picture analysis; the study showed that the Adenoma Detection Rate (ADR) increased tremendously after AI application compared with that in the traditional group. Therefore, it can be said that with this system, the polyp and tumor detection rate of colonoscopy have been well increased. On the other hand, Chen et al. (2020) and Wu L (2019) have developed a deep learning-based image analysis system for esophagogastroduodenoscopy (EGD), which can classify the stage of duodenal diseases. This system leads to an excellent reduction of the blind spot’s missed diagnosis rate, which is 5.9% and 3.4% missed rate.

### 6.4 Screening for other tumors

In a study by McGenity et al. (2024), an innovative deep learning-driven system for analyzing slice pathological images was introduced. This system demonstrated the capability to automatically diagnose and classify breast cancer. Impressively, the overall accuracy rate achieved a notable 83.1% when compared to pathological results, which are considered the gold standard. Whereas, Hossain et al. (2024) presented a chest CT processing system based on deep learning, achieving a sensitivity rate of 91% for metastasis detection, thereby enabling the automated screening of metastatic breast cancer. Mohamed et al. (2022) introduced a fully automated breast cancer detection system. Initially, the U-Net network is employed to automatically extract and segregate the breast area from the surrounding body, which is considered noise in the breast cancer detection model. This yielded an accuracy of 99.33%, sensitivity of 100%, and specificity of 98.67%. Lotter et al. (2021) introduced an annotation-efficient DL approach that attains state-of-the-art performance in fields like mammogram classification, showcasing average sensitivity enhancements of up to 14% for AI methods compared to mammography experts. Yoo et al. (2018) presented a deep learning-based ultrasound image analysis system, which elevated the screening sensitivity for thyroid cancer from 84% to 92.0%, and successfully achieved automated screening for both benign and malignant thyroid nodules. Masood et al. (2018) Constructed a lung CT image processing system using the Internet of Medical Things (IoMT) and DL, which successfully predicted the malignant transformation stage of pulmonary nodules, achieving a classification accuracy rate of 84.6%. Wu Yazhou and Chen (2022) and Zadeh Shirazi et al. (2021) employed a Deep Convolutional Neural Network (DCNN) as a semantic segmentation model for segmenting seven distinct tumor regions, achieving a segmentation accuracy of 70%.

### 6.5 Screening for eye diseases

Hassan et al. (2024) utilized DL techniques to process retinal images, enabling the automated screening and severity grading of diabetic retinopathy. The AI method exhibited a sensitivity and specificity of 100% and 88.4%, respectively, for diagnosing severe lesions. With the ability to obtain the sensitivity of 85.2% and specificity of 92% for lesions. Archana and Jeevaraj (2024) developed a DL-based system for processing the images of the eye, and it provided an outstanding AUC-ROC from 99.3% to 99.7% for cataract classification. This aids in conducting automated screening and collective management of cataract-affected persons. Lombardi et al. (2021) proposed a new deep learning system to predict brain age through native resting-state scalp EEG raw data and reached an accuracy of 89.7%. Wu et al. (2018) found in a study on the diagnosis of fungal keratitis, that automatic hyphae detection technology is sensitive at 89.3% and specific at 95.7%. The AUC value of the ROC curve is 94.6%, which is timely, accurate, objective, and quantitated to provide evaluation criteria for fungal keratitis.

### 6.6 Intelligent diagnosis

AI systems within healthcare provide patients with reliable and tailored services that go beyond traditional testing. They effectively assist patients in overseeing their individual wellbeing, tracking medical conditions, delivering treatments, offering psychological guidance, and handling dietary considerations. This holistic support contributes to extending patient longevity and enhancing their overall quality of life (Origimid, 2022). However, the effectiveness of the AI model is limited by the size of the training dataset. A model trained on one dataset may not perform optimally when applied to a different dataset with different characteristics. Therefore, it is important to incorporate an external test dataset during model training to evaluate its ability to generalize. Additionally, while many AI-driven diagnostic methods focus on analyzing medical imaging data, it is essential to recognize that clinically meaningful research outcomes require a holistic assessment of various patient indicators by healthcare professionals. Therefore, future research efforts should prioritize the comprehensive use of diverse clinical data to improve the effectiveness and wide-ranging applicability of AI models (Data sharing in the age of deep learning, 2023).

### 6.7 Diagnosis of infectious diseases

The outbreak of novel coronavirus disease (COVID-19) provides a suitable application opportunity for AI technology. AI technology has made a significant progress in the diagnosis, typing, risk prediction, and adjuvant treatment of COVID-19 (Ennab and Mcheick, 2022b). Shorfuzzaman et al. (2021) introduced a deep learning fusion framework that incorporates the principles of transfer learning, achieving intelligent diagnosis of COVID-19 patients with a classification accuracy of 95.5%. Quiroz et al. (2021) confirmed that the ML approach can be employed to automatically assess the severity of COVID-19, aiding in the classification and diagnosis of COVID-19 patients with an accuracy rate of 96.0%, a sensitivity of 84.5%, and a specificity of 92.9%. This enables the prioritization of follow-up diagnosis and treatment. Salem et al. (2023) developed a machine and deep learning system to identify metabolites and clinical features linked to gallstone disease. Pang et al. (2019) developed an innovative YOLOv3-arch model specifically designed to identify cholelithiasis and classify gallstones in CT images. This model significantly enhanced accuracy, achieving a 92.7% accuracy rate for identifying granular gallstones and an average accuracy of 80.3% for identifying muddy gallstones. However, it is important to note a major limitation of these models: healthcare workers cannot predict COVID-19 in patients with other underlying conditions such as COPD, hypertension, asthma, and more (Ennab and Mcheick, 2022b).

### 6.8 Diagnosis of medical diseases

As medical imaging technology advances and clinical diagnosis accuracy improves, clinical diagnostic approaches employing DL technology have experienced significant growth (Ennab and Mcheick, 2022b). Arbabshirani et al. (2018) and Kim et al. (2024), each constructed a DL-based brain CT image processing system, with accuracy values of (73%) and (84.6%), realizing automatic detection of acute neurological events such as stroke. Lo et al. (2021) suggested an automated ischemic stroke diagnosis method using deep DL, achieving a sensitivity of 98.1%, specificity of 96.9%, and an accuracy rating of 99.3%. This method effectively offers clinicians valuable diagnostic recommendations for acute ischemic stroke cases. Bibi et al. (2020) created a system that combines DL and the IoMT to achieve swift and secure identification and classification of leukemia, boasting an impressive average accuracy rate of 99.6%. This system facilitates real-time communication between healthcare professionals and patients regarding leukemia detection, diagnosis, and treatment, effectively conserving clinicians’ time and efforts. Jing et al. (2023) developed a DL system aimed at predicting the likelihood of recurrence and disability outcomes in patients who have experienced a transient ischemic attack or ischemic stroke. Eckardt et al. (2022) created a deep learning model specifically tailored for detecting NPM1 mutation and forecasting the presence of acute myeloid leukemia, attaining an impressive accuracy level of 95%. Bukhari et al. (2022) developed a deep learning framework for identifying leukemia cancer in microscopic blood samples by incorporating squeeze and excitation learning techniques, achieving an accuracy rate of 89.72%. Hamedan et al. (2020) employed Expert Systems (ES) to assess chronic kidney disease, integrating AI technology with artificial expert judgments. The results indicated that the ES significantly outperformed all other models in performance for predicting chronic kidney disease when the accuracy of the ES was 92.1%, sensitivity was 95.4%, and specificity was 88.9%.

### 6.9 Diagnosis of surgical diseases

Deep learning image knee analytical system was designed for knee injury diagnosing, e.g., injured knee estimation, such as anterior cruciate ligament and meniscus tears by Dratsch et al. (2024). Deep learning models in Siouras et al. (2022) related to MRI-associated knee injury estimation with accuracies ranging from 72.5% to 100%. The processing system based on deep learning for X-ray images was developed by Lindsey et al. (2018) to detect and localize fractures. After they integrated AI technology, the sensitivity in fracture detection of the clinicians improved from 80.8% to 91.5%, and the specificity increased from 87.5% to 93.9%, the CT image analysis system, designed by Fu et al. (2019), intended for automatic diagnosis and visualization of interfemoral fractures by identifying the most probable area of the fracture.

### 6.10 Adjuvant therapy

The goal of is to aid individuals with early invasive cancer in accurately predicting the personalized survival benefits, allowing them to make informed treatment decisions, without supplanting conventional treatments (Zheng et al., 2024). Adjuvant therapy relies on machine learning models and has the potential to enhance patient survival (Howard et al., 2020). Machine learning techniques can accurately predict patient outcomes under different treatment regimens by modeling the complex interactions between risk factors in a data-driven manner (Alaa et al., 2020).

### 6.11 Treatment decision support

Radiation therapy plays a pivotal role in the treatment of various tumors (Frisch et al., 2024). Within the treatment process, it is imperative to precisely outline the organ at risk (OAR) to provide guidance for radiotherapy and predict the patient’s prognosis (Hall et al., 2022). Bird et al. (2021) constructed ML model using a multi-center dataset, with the goal of establishing precise and broadly applicable radiotherapy protocols for rectal cancer. Through rigorous validation studies and regulatory approvals, this treatment decision support system mitigates prescription errors and augments existing error warning mechanisms (Yang et al., 2023). Rawson et al. (2021) constructed machine learning-driven systems for antimicrobial prescription decision-making, offering clinical decision support in antibiotic management. The AI-generated prescription recommendations have achieved a level of accuracy similar to that of clinicians. Bozyel et al. (2024) introduced an ML-based prescription recognition system to enable automated early detection and correction of prescription errors in heart disease patients, achieving a clinical effectiveness rate of 85%. Hooshmand et al. (2021) utilized DL techniques to explore potential COVID-19 drug candidates that exhibit minimal side effects and promising efficacy. Ke et al. (2020) employed DL methodologies to pinpoint existing drugs with the potential to combat COVID-19, ultimately discovering over 80 promising candidates for coronavirus treatment.

### 6.12 AI systems for medical surgical robots

Surgical robot technology stands as the foundational element of Computer Integrated Surgery (CIS). It brings quantification to the complete medical process, spanning from diagnosis and procedural planning to surgery, recovery, and observation (Lee et al., 2024). By integrating robotics and IT technology into the medical domain, it aims to enhance surgical procedures, making them safer and more efficient through rigorous objectification (Reddy et al., 2023). Surgical robots typically fall into two primary categories. This is identified to be the first category where robot systems are developed, which are designed to operate on body cavities such as the abdomen, chest, and lumbar regions. An illustrative example is the da Vinci robot system, which conducts surgery using a master-slave approach relying on 3D imaging (Biswas et al., 2023). The second category pertains to surgical robot systems designed for addressing lesions within the brain, bones, or muscles. These systems create a 3D model of the patient’s lesion using pre-existing medical images. The surgery planning, therefore, becomes a pointer of other factors like the lesion location, lesion condition, image information regarding the lesion, and the position of the patient in relation to the operating table, along with the 3D patient coordinate system realized before surgery (Chiou et al., 2022). Employing surgical robots yields several advantages, including reduced surgery duration, lowered risk of bleeding and infection for patients, and decreased fatigue and hand tremors for medical staff during procedures. Besides, it has benefits to hospitals by the patients having short stay lengths in hospital, therefore, increasing bed turnover, and positive patient outcomes through minimally invasive surgical technologies (Lee et al., 2024). At the moment, IR is used in broad clinical fields, such as orthopedics, biliary tract, throat, and liver surgeries, to mention but a few (Gumbs et al., 2021; Zhang and Zhang, 2023) used IR in spinal surgery in whose application brings out the accuracy in screw positioning and uses a few intraoperative fluoroscopies and has a low incidence in postoperative complications. Xie et al. (2021) used da Vinci surgical system in managing biliary cysts in infants below 1 year and brought out that the use of IR is safe in this clinical application. Arora et al. (2024) performed robotic surgery, in this case, transoral, for the extraction of masses in the throat, which was well exposed visually and that one encountered no major adverse reactions. Serednicki et al. (2023) used IR in liver surgery, and one enjoys the benefits of low blood loss and few adhesions, therefore short stay days in hospital and the duration for postoperative recovery is short.

## 7 The interpretability models

With the widespread adoption of AI solutions in healthcare, directly understanding models’ working mechanisms and opening the black box has become increasingly important (Pavlidis, 2024). Building confidence in machine learning models is now necessary for fully adopting AI systems. Thus, model interpretability is highly sought-after, especially in high-risk domains requiring dependability like healthcare (Ennab and Mcheick, 2022a). AI models in healthcare may provide contradictory results to medical institutions, potentially having disastrous consequences across cultures and situations. Interpretable approaches can clarify how a model choice is made, allowing tracking each output result and controlling model outputs (Longo et al., 2024). Interpretable AI models enable users to query, comprehend, correct, and even advance the learning system. Overall, interpretable learning models allow end-users like doctors to assess the model before taking any further action. By justifying predictions, interpretable machine and deep learning models give users the option to reject or accept the predictions and recommendations (Salih, 2022).

### 7.1 Types of the interpretability models

#### 7.1.1 Ante-hoc(Intrinsic)

The fastest way to easily interpret a model is designing it with an interpretable structure initially. A simple model like a decision tree is easy for humans to interpret by looking at its structure. A model with a simple structure was termed Intrinsic (originally equipped) because it already secured interpretability itself, also expressed as having transparency (Cao et al., 2024). The advantage of Intrinsic is explaining “how the model works.” However, due to the trade-off, intrinsic models have low accuracy (Carvalho et al., 2019).

#### 7.1.2 Post-hoc

If the model itself lacks explanatory power, its prediction results must be interpreted *post hoc*. Most interpretability techniques in machine and deep learning are *post hoc*. Ideally, the model would have high accuracy and explanatory power, but this is rare in reality (Cao et al., 2024). Complex high-performing models are commonly used, with *post hoc* analysis done. Post-hoc examples are explained with the viewpoints introduced later (Moradi and Samwald, 2021).

#### 7.1.3 Global

The global technique describes all model predictions based on understanding its logic, or at the module level, scopes describing how one module affects predictions (Carvalho et al., 2019).

#### 7.1.4 Local

Local techniques describe specific decisions or predictions. A range describing a prediction group by bundling predictions is also a local technique. Compared to global methods, local techniques have a smaller scope to explain, making them relatively feasible and inexpensive. Additionally, even if the overall prediction trend is unexplained, one or a few predictions can be described nearly perfectly (Moradi and Samwald, 2021).

### 7.2 The interpretability models

The categorization of the interpretability models may vary slightly depending on specific implementations and use cases as shown in Table 2.

**TABLE 2:** Categorization of the interpretability models.

Interpretability model	Public	Local	Post-hoc (model-agnostic)	Ante-hoc (model-intrinsic)
The interpretability based		√	√	
Bayesian Nonparametric		√		√
GAM	√			√
MAPLE	√		√	
Anchors	√		√	
Perturbation-Based Methods		√	√	
Attention Based		√	√	
SHAP	√	√	√	
Grad-CAM		√	√	
Textual Justification		√	√	
LIME		√	√	
Testing Concept Activation Vectors		√	√	
Similar Images		√	√	

**TABLE 3:** Limits of the interpretability models.

Model	Limits
The interpretability based	Offers detailed local explanations but may overlook the overall model behavior. The explanations can change based on the selected samples during analysis
LIME	Relies on data samples tailored extensively to estimate the local model. Explanations can differ with sample selection, leading to inconsistent results. High computational overhead
SHAP	Calculation of Shapley values is intensive, especially with high dimensionality. Based on cooperative game theory assumptions, which might not always hold
Grad-CAM	Focused on Convolutional Neural Networks, might not be effective for other models. Provides class activation maps but lacks deep feature-level explanations
Bayesian Nonparametric	Designed for CNNs, not useful for other model architectures. Provides class activation maps but lacks detailed feature-level explanations
Generalized Additive Models (GAM)	Cannot capture complex interactions between features, less expressive power. Assumes linear and additive relationships, which might not hold in reality
MAPLE	Provides local-level explanations at the expense of the global view. Explanations can vary depending on the samples used during analysis
Anchors	Powerful for black-box models, less effective for transparent models. Provides discrete explanations, which might not capture model subtleties
Perturbation-Based Methods	Computationally expensive for complex models. Explanations may vary due to random sampling, affecting stability
Attention-Based Methods	Difficult to implement and complex. Provides importance to input data parts but lacks clear feature explanations
Testing Concept Activation Vectors	Designed for neural networks, limited applicability to other models. Understanding results requires deep learning expertise
Similar Images	Effectiveness limited by dataset quality. Provides feature-level information but explanations are image-level
Textual Justification	Effective only if people can understand and believe them. Can potentially bias or leave ambiguous text explanations

#### 7.2.1 The interpretability-based model using the relative weight of the image features

Ennab and Mcheick (2022a) presented an interpretability-based model using statistics and probability principles to train datasets by determining the relative weights of variables indicating their respective significance in predicting and estimating disease likelihood. The variables are either patient symptoms or characteristics of injured organ regions in medical images. Dividing each variable’s weight by the sum of all weights gives the relative weights. Training the dataset determines infection likelihood. Data were collected as previously described in Mohammad Ennab and Hamid Mcheick (22 October 2022).

#### 7.2.2 LIME (local interpretable model-agnostic explanation)

LIME is a model-agnostic technique explaining which features are most important in a feature space region (Shi et al., 2020). LIME’s core idea is computing a Local Surrogate Model in a region of interest, which is an easily interpretable model like linear or decision tree trained to mimic a complex model’s behavior (Garreau and von Luxburg, 2020). For an explanation, LIME creates new similar data points with slightly altered values. Feeding the perturbed points into the complex model reveals relationships between perturbed features and predictions, captured by the surrogate model (Garreau and von Luxburg, 2020).

#### 7.2.3 SHAP

SHAP uses game theory to measure each attribute’s impact on the prediction process. The Shapley value evenly divides advantages among contributing parties (features) when contributions are unequal (Shapley, 2016). In other words, Shapley values are based on features interacting to influence predictions toward a value. It attempts to evenly distribute contributions across all feature subgroups. Specifically, the Shapley value uniformly distributes the difference between the prediction and average prediction among the instance’s feature values needing explanation (Strumbelj and Kononenko, 2011). SHAP values provide a unique additive feature importance measure satisfying attribution features (local accuracy, missingness, consistency). These features represent intuitive rules for determining the final prediction, translatable to the machine learning problem. However, direct Shapley value computation requires efficient computation, needing to check every permutation combination (Mitchell et al., 2022).

#### 7.2.4 Grad-CAM

Class Activation Mapping (CAM) is a technique widely used in computer vision to create visual representations displaying contributions of different image regions to neural network predictions (Selvaraju et al., 2017). This produces a heatmap-resembling image with each pixel signifying the activation level for a class. Pixel values range from 0 to 1, typically shown as a 0 to 255 grayscale image. Higher scores indicate regions in the original image having a stronger influence on the network’s response or prediction. Overlaying CAM onto the original image enhances visual appeal and informativeness. Unlike CAM requiring model structural changes and retraining, Grad-CAM utilizes pretrained weights to backpropagate gradients to the desired parameter layer (like convolutional) when predicting an image (Schöttl, 2020). This yields a gradient matrix with identical dimensions to the parameter layer’s output feature map. By globally average pooling the gradient matrix across spatial dimensions, a vector with equal length to the feature map’s channel count emerges. This vector contributes to weighing the diverse feature map channels, ultimately creating a heatmap visualization. As Grad-CAM avoids model architectural adjustments and retraining, it offers a more flexible, efficient CAM alternative (Liu et al., 2023). Conventionally, a neural network’s classification module uses a fully connected model processing extracted features, converting them into class probability scores via a softmax layer. The highest scoring class then dictates the ultimate prediction. Grad-CAM takes a different approach by not just discriminating between classes but also pinpointing relevant image regions. This is achieved by exploiting gradients (derivatives) from the final convolutional layer’s feature map (Vinogradova et al., 2020). These gradients serve as a tool to emphasize important areas significantly impacting the eventual prediction.

#### 7.2.5 Bayesian nonparametric model

Guo et al. (2018) created a Bayesian nonparametric approach to build a parameter space with infinite dimensions. In other words, model size can fluctuate in response to data increases or decreases, determined by the number of data parameters used. It requires few assumptions to learn data and perform clustering. Growing data can also be continuously aggregated into proper classifications, this model also makes predictions concurrently. A spatial data model comprises all issue-related properties manageable based on the unique learning problem (Ribeiro et al., 2018). The core of the non-parametric Bayesian statistical model is setting the data probability distribution function to an arbitrary, flexible distribution rather than a specific parametric one and placing a prior distribution on this to perform posterior inference. In Bayesian statistical models, non-parametric modelling allows flexible modelling of data distribution, random effect distribution, or parameter of interest prior distribution, presenting a wider probability model class. Thus, the prior distribution for random distribution is key in nonparametric Bayesian statistical modelling, with the probability most frequently used (Noh et al., 2014).

#### 7.2.6 GAM

Kraus et al. (2024) introduced GAM, a generalized additive global variable weight technique considering neural network swarm forecast patterns. GAM’s global interpretation describes the neural network’s non-linear representation. GAM also enables modifying subpopulation granularity and tracking global interpretations for particular samples. In statistics, a generalized additive model (GAM) is a linear model where predicted variable values are the aggregation of several unknown smooth functions defined for the predictors. The purpose is inferring a predictor smooth function whose aggregate composition approximates it. This structure is easily interpretable, allowing the user to see each variable’s importance, i.e., its effect on the predicted output via its function (Linardatos et al., 2021).

#### 7.2.7 MAPLE

The key difference between using MAPLE as a black-box model explanation versus a predictive model is fitting MAPLE to the black-box model’s prediction in the first case and the response variable in the second. Since MAPLE is a very accurate predictive model providing correct predictions, it avoids trading off performance and interpretability. It finds global trends using local examples and explanations. MAPLE differs from other frameworks by its training distributions (El Shawi et al., 2019). Data were collected as previously described in Mohammad Ennab and Hamid Mcheick (22 October 2022). Maple alters tree ensembles to provide local explanations that detect global trends and example-based explanations. It uses the ensemble to determine the most important training points for a new prediction, building a linear model from those points for prediction and local explanation (Plumb et al., 2018).

#### 7.2.8 Anchors

Anchors is a model-independent, rule-based local explainer approach (Ribeiro et al., 2018). Anchors ensure that projections from the same anchor are roughly equal. Anchors, in other words, identify the features that are sufficient to correct the forecast while adjusting the others that do not influence the prediction. The bottom-up strategy, in which anchors are constructed sequentially, is one type of anchor building (Ribeiro et al., 2018). Data were collected as previously described in Mohammad Ennab and Hamid Mcheick (22 October 2022). Anchor describes individual predictions for black-box classification models by finding decision rules that sufficiently “anchor” the predictions. A rule freezes a prediction if changes in other feature values do not affect the prediction. Anchor leverages reinforcement learning techniques with graph search algorithms to reduce the number of model calls (required running time) to a minimum while still being able to recover from the local optimization (T, 2020).

#### 7.2.9 Perturbation-based methods

Perturbation is the most basic technique for assessing input property modifications on output. This involves eliminating, masking, or altering specific inputs, then forward passing output and comparing to the original output. This is similar to the sensitivity analysis performed in parametric control system models. The input features that have the greatest impact on the outcome are prioritized. Because a forward pass must be performed after perturbing each set of features in the input, it is computationally intensive. In the case of picture data, the perturbation is accomplished by covering areas of the image with a grey patch, thereby obscuring them from the system’s view (Singh et al., 2020). In images, disturbance is done by covering areas with a grey patch, concealing them from the system’s view (Ennab and Mcheick, 2022a). The perturbation-based methods are broadly divided into input sampling-based methods and input optimization methods. RISE (Randomized Input Sample for Explanation) is the most representative algorithm among input sampling-based methods that put a random mask on the input image and saves the AI output. After around 8,000 repetitions, a linear combination of random masks and AI output values is performed. In this way, random masks made with high output values show consistency in feature exposure, allowing for a proper explanation (LG AI Researcher, 2021). However, the problem with this approach is that it requires about 8,000 outputs to illustrate. Another limitation is that the results are different each time it is performed on the same input image because a random mask is used (LG AI Researcher, 2021). The most representative algorithm in the latter input optimization method is an algorithm called extremal perturbation, which finds feature parts showing high output values by the optimization method. The problem with this approach, however, is that it relies only on the optimization method. When an accurate solution is obtained, a very interpretable explanation can be obtained, but when an exact solution is not obtained, features that seem completely unrelated may be displayed. In addition, since it uses a numerical optimization method, it has a computation time problem (LG AI Researcher, 2021).

#### 7.2.10 Attention based

The fundamental concept of attention is motivated by how people pay attention to various areas of an image or other data sources in order to interpret them. The technique employed attention mechanisms, which included an image model and a language model, to show the detection process (Li et al., 2021). The interpretation in attention-based involves combining mechanisms for the selective traits of dominant features with attention towards the reported trait. One part of this will be selecting some of the hidden states over the time steps and on top of that, adding an attention layer to the present deep learning model. An attention score is computed at each important feature or time sequence to denote its importance. The attention mechanism found in the language model was used to learn the mapping between diagnostic reports and sights (Singh et al., 2020).

#### 7.2.11 Testing concept activation vectors

In Kim et al. (2017), Concept Activation Vectors (TCAV) is another innovative approach used to explain the acquired characteristics of successive layers in terms of human-understandable concepts to domain experts without deep learning comprehension. It uses the network’s directional derivative in concept space in a similar way that saliency maps use input feature space. The directional derivative of the network in concept space is treated as a saliency map in TCAV. In this way, TCAV is perfectly suitable as the approach for detection of microaneurysms and aneurysms in the retina when saliency maps are used for the classification of the Diabetic Retinopathy (DR) level prediction and provides understandable reasoning to the physician for the level of DR. This provides justification whether a conceptual or physical structure is present in the image (Singh et al., 2020). However, many clinical concepts in medicine, such as structural texture or tissue shape, cannot be fully described by TCAV directly to prove their existence or non-existence (Kim et al., 2018). Many clinical concepts, such as form, texture, or shape, cannot be effectively described in terms of presence or absence and require a continuous scale of assessment (Ennab and Mcheick, 2022a).

#### 7.2.12 Similar images

Stano et al. (2020) proposed research assessing layers of a 3D-CNN using a Gaussian mixture model (GMM) and binary encoding of training and test pictures based on their GMM components to offer explanations for comparable 3D images. As an explanation for its result, the software returned activation-wise similar training pictures utilizing the atlas. The model includes a perceptual code in binary vector space that defines the CNN’s processing of the input sample (Kim et al., 2018). A collection of the most perceptually similar and dissimilar samples may be retrieved from an existing atlas of labelled samples in order to support and further explain the choice made by the CNN model by calculating distances between pairs of samples in this perceptual encoding space. Applications of this model include Computer-Aided Diagnosis software using Computed Tomography (CT) data from medical imaging tests (Stano et al., 2020). The same imaging model was carried on the 3D MNIST datasets and Magnetic Resonance Imaging (MRI) datasets, and the findings were congenial atrophy conditions. There is an indication in some of the cases that the breakthrough of similarity with activation is invariant to the picture spatial orientation, and impact may be there in choices related to pictures returning (Singh et al., 2020).

#### 7.2.13 Textual justification

The justification for the choice was given in the form of words or phrases and, on general, can communicate directly to both expert and non-expert users (Singh et al., 2020). A diagnostic phase and visual heatmaps for the breast-mass classification was developed using an explanation model that took input from the visual features of a classifier as well as the prediction embeddings (Lee et al., 2018). Justification generator was trained to generate justifications in the existence of a small number of medical reports using a visual word constraint loss (Singh et al., 2020). Data were taken as described earlier in Mohammad Ennab and Hamid Mcheick (22 October 2022). A diagnostic network and a justification generator are the two parts of the overall design of the model. Any general CADx network (classifier of malignant mass and benign mass) can be used as the diagnostic network. A visual characteristic and a diagnosis made by the diagnosis network are used by the justification generator. A visual word constraint loss is developed in the training stage to efficiently train the justification generator by preventing the training set’s sentences from being duplicated (Lee et al., 2019).

### 7.3 Characteristics of the interpretability models

It is important that when choosing the interpretation models to implement, many-paged features should be carefully and perhaps realized that no one fits all solution may exist:

1. Model complexity vs. model interpretability: Some interpretability method simplifies complex models to be interpretable. Still, in that simplification, the majority of the time, a tradeoff between model accuracy and interpretability are made (Johansson et al., 2011).
2. Suitability to different model types: not all interpretability method has a similar level of suitability for different kinds of machine learning models. Some work well with large deep neural networks, while others work better for decision tress or linear (Escalante et al., 2018).
3. Local explanations: Most of the interpretation methods aim to present local explanations: for each prediction, the user can get an idea of which features that have played into account for that particular output. That is to say that these methods do not aim to have a global perception of the model’s behavior (Linardatos et al., 2020).
4. Consistency: The interpretability methods can explain why the explanations are consistent depend on the way the input samples are chosen and hence might produce different explanations for marginally different sampled data points (Hu et al., 2019).
5. Discrete explanation: Some of them offer differences in terms of interpretation where they provide interpretations in a discrete or rule-based way; therefore, the fine-grained nature of the original model might miss this kind of methods (Gilpin et al., 2018).
6. Interpreting complex models: On the other hand, the interpretation of complex models like deep neural networks might be quite troublesome, and yes, the explanation might be done (Carvalho et al., 2019).

Table 3 summarizes the limitations associated with various interpretability models. Each model offers distinct advantages and challenges, with trade-offs between local and global explanations, computational overhead, and applicability across different architectures. While some models (like LIME and SHAP) provide local insights at the cost of consistency, others (like Grad–CAM and Bayesian Nonparametric) are tailored for specific use cases, such as convolutional neural networks. The table highlights how different interpretability methods suit various needs but may have constraints related to stability, complexity, and applicability beyond specific models or domains.

### 7.4 Assessment of the interpretability models

The interpretability models can mainly be classified under the qualitative and quantitative models. The models that are applied to the quantitative evaluation are as follows:

1. KAR method (keep and retrain): the modularity of the analysis on how the removal of the least significant N % pixel features in the saliency map is done in respect of the change in retrained model accuracy (Kim et al., 2019).
2. ROAR method (remove and retrain): the removability analysis of how the accuracy of the retrained model is affected when excluding the most important N % pixel features in the saliency map (Kim et al., 2019).

The following are used as measures of qualitative evaluation:

1. Coherence: This means that the input pattern that is closely related to the prediction given by the interpretability method needs to have an attribute that is somehow (Gilpin et al., 2018).
2. Selectivity: in the case that input image, the exclusion of pixels that are rated important in the saliency map by the method, this, in turn leads to reduction of related probability that is related to the model prediction category that corresponds (Fuhrman et al., 2022).
3. Implementation invariance: In the case of two models, for explanation, being similar, that is to say, two models that provide the same input produces the same output, then this, in turn means that interpretability method should give the same for two models (Carvalho et al., 2019).
4. Class sensitivity: The explanation that is generated by interpretability method should be sensitive to the category (Nielsen et al., 2022).
5. Explanation continuity interpretability methods should give similar explanations to similar input (Longo et al., 2024).

**FIGURE 3:**
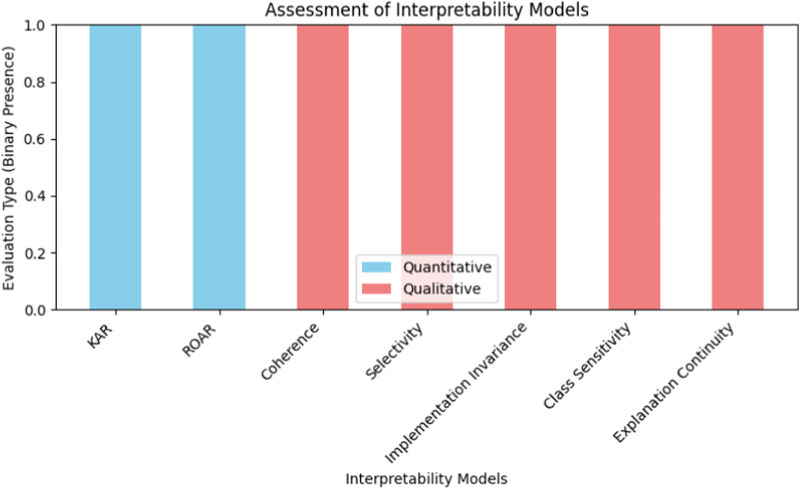
Assessment of interpretability models categorized into quantitative and qualitative evaluation methods.

Figure 3 explains the categorization of the interpretability models into quantitative and qualitative methods. The quantitative models (KAR and ROAR) focus on performance changes with model retraining based on feature importance. In contrast, qualitative models (such as Coherence, Selectivity, Implementation Invariance, Class Sensitivity, and Explanation Continuity) assess interpretability based on the behavior and consistency of explanations across different inputs and predictions.

### 7.5 Recapitulation of main points

The application of AI in healthcare has demonstrated immense potential in automating diagnosis, treatment decisions, and patient monitoring. However, several key challenges persist:

• Advantages: AI systems have shown remarkable accuracy in tasks like diagnostic imaging, risk prediction, and disease screening. The ability of deep learning models to process large datasets quickly and accurately has outperformed traditional methods in several areas (e.g., cancer detection and diagnostic imaging).
• Limitations: The black-box nature of many deep learning models makes it difficult for healthcare providers to trust and interpret their decisions. The lack of transparency and interpretability remains a critical barrier to widespread adoption. Additionally, AI models often struggle to generalize across diverse patient populations, leading to reduced performance in real-world clinical settings.
• Challenges: Another significant challenge is the computational cost and real-time applicability of certain models. Many AI systems require significant computational resources, which limits their deployment in resource-constrained healthcare facilities. Furthermore, the integration of uncertainty quantification with interpretability models remains an underexplored area that could provide more reliable and explainable AI systems.

## 8 Lessons learned and future directions

Through our systematic review, several trends and common issues have emerged:

• Lack of real-time interpretability: Most high-accuracy AI models operate as black boxes, which limits their clinical applicability.
• Trade-offs between accuracy and interpretability: Models that prioritize accuracy often do so at the expense of interpretability, and *vice versa*.
• Generalizability issues: The majority of studies focus on specific datasets, making it difficult to generalize their findings to broader clinical populations.

### 8.1 Future directions

To address these challenges, future research should focus on developing hybrid models that balance accuracy and interpretability. Additionally, incorporating uncertainty quantification methods can improve the reliability of AI models in healthcare. Additionally, as AI continues to evolve, several key areas require further investigation:

• Improved Interpretability: Future research should focus on developing models that balance both accuracy and interpretability. Techniques such as Local Interpretable Model-Agnostic Explanations (LIME) and SHAP (Shapley Additive Explanations) could be further explored to enhance the transparency of deep learning models. Additionally, more effort is needed to integrate uncertainty quantification techniques to improve model reliability in real-time clinical settings.
• Incorporation of Multimodal Data: AI systems that can process diverse types of clinical data, such as medical imaging, patient history, and genetic information, are likely to provide more comprehensive and accurate predictions. Research should explore how multimodal AI systems can improve patient outcomes by integrating various data sources.
• User-Centered Design: A key challenge in AI healthcare applications is the need for user-centered design. Future research should explore how AI models can be developed in collaboration with healthcare providers to ensure that they are user-friendly, interpretable, and aligned with clinical workflows.
• AI in Personalized Medicine: While this review focused primarily on diagnostic imaging, future research should explore how AI can be applied to other domains, such as genomics and personalized medicine. AI models that can predict patient-specific outcomes and suggest personalized treatment plans based on genetic data have the potential to revolutionize healthcare.
• Learning-Based Manipulation: In the context of learning-based manipulation, as discussed in Stefanelli et al. (2023), future research should investigate how AI systems can integrate multiple sensory inputs (e.g., force, temperature) to mimic human-like responses in medical robotics and prosthetics. Testing and refining these methodologies in clinical scenarios could lead to more adaptive and responsive AI-driven prosthetic systems.

## 9 Conclusion

This review systematically examined AI models in healthcare, focusing on the trade-offs between accuracy and interpretability. We highlighted the most commonly used models and metrics, and identified key challenges, such as the black-box nature of deep learning models and generalizability issues. Our findings suggest that future AI research should prioritize transparency and safety, particularly in high-risk healthcare applications. By addressing these issues, healthcare providers can develop AI solutions that not only enhance performance but also build trust among stakeholders, ultimately leading to safer and more effective patient care. Additionally, incorporating strategies like those presented by Stefanelli et al. (2023), which integrate force and temperature information in prosthetic control, could offer new avenues for enhancing AI systems’ human-like capabilities in healthcare.
